# Single-stage surgical repair of airway gastric fistula after esophagectomy

**DOI:** 10.1186/1749-8090-9-30

**Published:** 2014-02-08

**Authors:** Hui Shi, Wen-Ping Wang, Qiang Gao, Long-Qi Chen

**Affiliations:** 1 Department of Thoracic Surgery, West China Hospital, Sichuan University, No. 37, Guoxue Alley, Chengdu 610041, China

**Keywords:** Airway gastric fistula, Esophageal cancer, Esophageal surgery, Reoperation

## Abstract

Airway gastric fistula (AGF) is a rare but catastrophic complication after esophagectomy. Surgical repair with viable tissue interposed between the airway and alimentary tracts remains the definitive treatment. However, it is challenging for surgeons, and only anecdotally described in sporadic case reports due to the complexity of the techniques necessary for successful surgical intervention. Here, we report two cases successfully managed via single-stage surgical re-exploration. On outpatient follow-up, the two Chinese patients were progressing satisfactorily without complaint of any dyspnea or dysphagia.

## Background

Airway gastric fistula (AGF) is a rare but catastrophic complication after esophagectomy for esophageal cancer with an occurrence of 0.3 % to 1.9 % [1]. Definitive treatment is controversial due to the rareness of this entity. Nonoperative management, including endoscopic stents implantation is increasingly applied, though it is usually considered for a small fistula with minimal clinical symptoms. When there is gastric tube necrosis, bronchial gangrene, or incomplete control of the fistula with persistent mediastinal abscess, surgical repair may represent a reasonable salvage procedure for acceptable patients. Howerver, surgical re-exploration is challenging for surgeons because of the high invasiveness and mortality, thus it is only anecdotally described in sporadic case reports. Here, we presents two cases of AGF successfully managed by single-stage surgical repair, one in the early postoperative period and the other one late in the follow up of the patient. They are different in physiopathology and management.

## Case presentation

### Patient one

**Figure 1:**
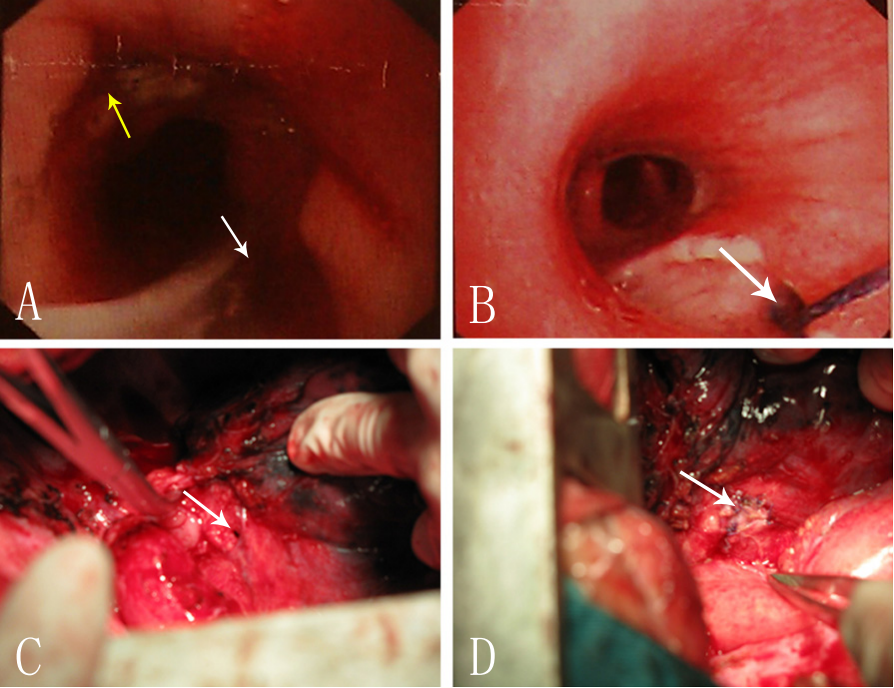
**Preoperative endoscopy and intraoperative view of patient 1. (A)** Esophagogastroscopy showing the anastomotic stoma (yellow arrow) and a fistula between anastomotic stoma and right intermediate bronchus (white arrow). **(B)** Bronchoscopy showing fistula (arrow head) within the right intermediate bronchus surrounded by large mucosa erosion. **(C)** Intraoperative view of an oval fistula (arrow head) in the right intermediate bronchus after resection the diseased gastric tube. **(D)** Intraoperative view of the repair of right intermediate bronchial defect with a pedicled pericardial flap using interrupted suture (arrow).

A 50-year-old Chinese man with a 3-week history of progressive dysphagia was diagnosed with a squamous cell carcinoma of the lower esophagus. He underwent a esophagectomy and 2-field lymphadenectomy through a right thoracotomy. The first postoperative week progressed smoothly. However, he experienced mild coughing with aspiration while given clear liquid diet on the 8^th^ postoperative day. Although immediate esophagogram showed no evidence of anastomotic leakage, an esophagogastroscopy revealed an approximately 3-cm ischemic mucosal area covered with exudate beneath the anastomosis (Figure 1A). A further examination with fiberoptic bronchoscope showed a necrotic defect located about 2 cm below orifice of the intermediate bronchus (Figure 1B). On the basis of these observations, an anastomotic leak and a broncho-gastric fistula were diagnosed. At this time, The patient was not in good condition with serious mediastinitis, prompting emergent exploration after a multidisciplinary consultation.

He was brought back to the operating room for an exploratory thoracotomy. Mediastinal abscess was debrided, and the gastric conduit was taken down. Then, an oval defect, measuring 2 × 6 mm, with necrosis of the membranous wall of the proximal 2 cm of the intermediate bronchus was found (Figure 1C). All necrotic tissues of the stomach including the anastomosis site were excised completely to the point of exposure of the intact mucosa. Airway defect was repaired using a pedicled pericardial flap and absorbable polydioxanone 4–0 interrupted sutures (PDS; Ethicon, USA). Esophagogastric continuity was restored with an end-to-end anastomosis using a circular stapler (CDH25, Ethicon, USA)(Figure 1D). Over the ensuing days, the patient made a slow recovery in the intensive care unit. On the 12th day after reoperation, sips of water were allowed after confirming the absence of anastomotic leaks via esophagogram, and a full liquid diet was implemented. At 1 month he was discharged from the hospital without any respiratory or swallowing problems. At 1-year follow-up he was symptom free, breathing and eating regularly.

### Patient two

**Figure 2:**
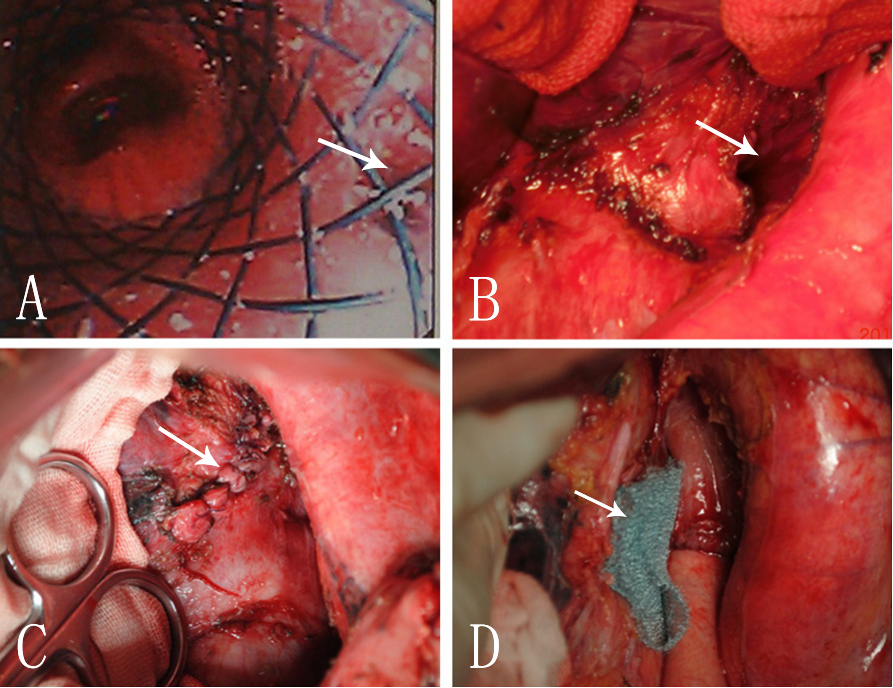
**Preoperative endoscopy and intraoperative view of patient 2. (A)** Bronchoscopy showing the unhealed fistula (arrow) 40 days after the second airway stenting treatment. **(B)** Intraoperative view of a large defect of left main bronchus (arrow) after resection the diseased gastric tube. **(C)** Intraoperative view of the reconstruction of left main bronchus with adjacent intrathoracic gastric tissue by use of an interrupted suture (arrow). **(D)** A PGA sheet (NEOVEIL) (arrow) was interposed between the reconstructed airway and alimentary tract.

A 48-year-old Chinese male patient was admitted to our hospital for the purpose of repair of a AGF. One year ago, he was diagnosed with lower esophageal cancer and underwent radical esophagectomy via left thoracotomy. Post-operative diagnosis was well differentiated squamous cell carcinoma, T2N1M0, Stage IIB. His initial postoperative course was uneventful. An esophagram showed no evidence of leak, and then he was given a regular diet. One month following the operation, he experienced a cough productive of gastric contents. Further evaluation by bronchoscope revealed a large AGF in the left main bronchus (Figure 2A). Then in the following months he was treated with 4 successive endoscopic approaches: clip application, fibrin glue injection, and 2 covered stent placement (Figure 2A). All of them failed and even increased the size of the AGF. The patient had developed serious aspiration pneumonia and thus was referred to our department for surgical treatment.

His general condition remained good, and he was taken to the operating room for definitive repair. The fistula was approached through a left thoracotomy along the original incision. The gastric conduit was remobilized and resected around the fused fistula beyond 2 cm, to ensure the blood supply of the residual gastric tissue which was to be used as a patch. A fistula measuring 1 × 3 cm was then revealed (Figure 2B), and the bronchial wall was reconstructed with the residual gastric tissue by use of an interrupted suture (4–0 Polydioxanone Suture, Figure 2C). A new gastric conduit was redone in the remnant stomach, an esophagogastrostomy was re-created with a circular stapler (CDH25, Ethicon, USA), and a polyglycolic acid (PGA) NEOVEIL^®^ sheet was interposed between the airway and the new gastric tube (Figure 2D). Postoperatively the patient did well. An esophagram showed no evidence of a leak or stricture. Oral feeding was resumed and he was discharged home 15 days after operation. On outpatient follow-up, he remained asymptomatic, with a measured weight gain, 20 months after the successful fistula repair.

## Discussions

Fistula between the airway and the gastric conduit following esophagectomy has been reported both early and late in the postoperative course. Early fistulas is related to postintubation injury, tracheobronchial erosion by gastric staple line, or an unrecognized thermal or blunt injury to the membranous airway. Late fistulas have been associated with chronic contained anastomotic leaks, cancer recurrence, and dilation of an anastomotic stricture. Prompt diagnosis is essential for successful treatment.

Definitive treatment of AGF after esophagectomy is challenging and influenced by the location, size and the pathogenesis of the fistula, and also by the presenting symptoms and the accompanying comorbidities of the patient. Over the past years, various management strategies have been described including conservative, endoscopic and surgical treatment [1,2]. In general, there has been a tendency to select non-operative treatments, in which endoscopic stents were most commonly used. Nevertheless, the location of the fistula did not always lend itself easily to a stent. What is more, the radical force provided by such stents may be detrimental to the tissue surrounding the fistula and result in enlargement of a fistula [1,3,4].

Surgical re-intervention, though fraught with high morbidity, would probably be the optimum radical treatment especially for large fistula or fistula with severe symptoms. Debridement of the septic focus, resection of the fistula, reconstruction of the airway and alimentary tracts, and interposition of various material if necessary were useful [5,6]. In case 1, direct closure of the bronchus was not possible because of friability of the membranous airway. We therefore interposed a pedicled pericardial flap. In case 2, the adjacent gastric wall was used to build a protective patch onto the tracheal defect through interrupted suture. Additionally, some studies have demonstrated that NEOVEIL^®^ sheet can be used as a covering material to prevent postoperative perforation after endoscopic submucosal dissection, while here it was used as a interposition between gastric tube and bronchus [7].

If operative risk was believed to be too great owing to the instable general condition of a patient, an endoscopic stent may be temporarily used to avert ongoing contamination of the airway from gastric contents. When improvement of systemic condition occurs, a surgical re-intervention should then be considered.

## Conclusions

Management of AGF requires the multidisciplinary knowledge and individual strategy. Although promising effective therapy is not established, it is unquestionable that aggressive surgical repair produces survival benefit for properly selected patients. Early and proper diagnosis with endoscopy, control of pulmonary and mediastinal infection, aggressive consideration of surgical intervention, complete resection of the fistula and restoration of functional airway and alimentary tracts are the keys to achieving a better outcome in acceptable patients.

## Consent

Written informed consent was obtained from the patient for publication of this case report and any accompanying images. A copy of the written consent is available for review by the Editor-in-Chief of this journal.

## Abbreviations

AGF: Airway gastric fistula.

## Competing interests

The authors have no commercial association or sources of support that may pose competing interests.

## Authors’ contributions

HS wrote the draft of the manuscript and obtained the written consent. WPW and QG participated in the manuscript writing and helped to the final writing of the paper and gave final approval of the manuscript. LQC participated in the manuscript revision. All authors have read and approved the final manuscript.
